# Exploring changes in integration, differentiation, rules, coordination and performance following the introduction of a hospital planning centre: a case study

**DOI:** 10.1108/JHOM-10-2021-0375

**Published:** 2022-04-08

**Authors:** Annelies van der Ham, Arno Van Raak, Dirk Ruwaard, Frits van Merode

**Affiliations:** Department of Health Services Research, Faculty of Health, Medicine and Life Sciences, Care and Public Health Research Institute (CAPHRI), Maastricht University Medical Centre + , Maastricht, The Netherlands

**Keywords:** Hospital planning, Integration, Coordination mechanisms, Rules, Performance

## Abstract

**Purpose:**

This study explores how a hospital works, which is important for further enhancing hospital performance. Following the introduction of a Hospital Planning Centre (HPC), changes are explored in a hospital in terms of integration (the coordination and alignment of tasks), differentiation (the extent to which tasks are segmented into subsystems), rules, coordination mechanisms and hospital performance.

**Design/methodology/approach:**

A case study was conducted examining the hospital’s social network, rules, coordination mechanisms and performance both before and after the introduction of the HPC. All planning and execution tasks for surgery patients were studied using a naturalistic inquiry and mixed-method approach.

**Findings:**

After the introduction of the HPC, the overall network structure and coordination mechanisms and coordination mechanisms remained largely the same. Integration and certain rules changed for specific planning tasks. Differentiation based on medical discipline remained. The number of local rules decreased and hospital-wide rules increased, and these remained largely in people’s minds. Coordination mechanisms remained largely unchanged, primarily involving mutual adjustment and standardization of work both before and after the introduction of the HPC. Overall, the hospital’s performance did not change substantially. The findings suggest that integration seems to “emerge” instead of being designed. Hospitals could benefit, we argue, from a more conscious system-wide approach that includes collective learning and information sharing.

**Originality/value:**

This exploratory study provides in-depth insight into how a hospital works, yielding important knowledge for further research and the enhancement of hospital performance.

## 1. Introduction

Integration in organizations, i.e. the coordination and alignment of tasks, is widely promoted as a means of improving hospital performance ( Drupsteen *et al.* , 2013 ; Kodner and Spreeuwenberg, 2002 ; Lega *et al.* , 2013 ). There is consensus that concepts such as supply chain management, lean strategies and other operations management theories can contribute to the improvement of hospital performance ( Aronsson *et al.* , 2011 ; Borges *et al.* , 2019 ; De Vries and Huijsman, 2011 ; Litvak *et al.* , 2008 ; Ludwig *et al.* , 2010 ; Van Merode *et al.* , 2004a , b ; Villa *et al.* , 2009 ). This is considered important to meet a broadly felt need to improve the quality, accessibility and affordability of healthcare systems ( Borges *et al.* , 2019 ) in general and hospitals in particular, which form a major cost item in the healthcare system ( Morgan and Astolfi, 2015 ).

A previous scoping study ( Van der Ham *et al.* , 2018 ) highlighted the fragmentation of research on logistics and operations in hospitals. Studies typically focus on one or two logistical parameters, specific logistical flows (patients, material or staff) or specific departments, as opposed to the hospital as a whole. This may be problematic; Ludwig *et al.* (2010) found evidence that in well-performing hospitals, departments cooperate for the benefit of the hospital’s efficiency, while efficient departments by themselves do not necessarily contribute to the overall efficiency of the hospital.

Lawrence and Lorsch (1967) indicate that differentiation is essential in order for integration to be effective. They define integration as “achieving unity of effort among the various subsystems in the accomplishment of the organization's task” ( Lawrence and Lorsch, 1967a , b , p. 4), whereas differentiation refers to “the state of segmentation of the organizational system into subsystems” ( Lawrence and Lorsch, 1967 , pp. 3–4). From this perspective, well-performing hospitals with sufficient departmental cooperation are likely to have the appropriate degree of integration as well as differentiation.

Given that understanding how a hospital’s logistical system works in practice is a first and necessary step towards improving how hospitals function, two case studies were previously conducted. In the first case study ( Van der Ham *et al.* , 2020 ), integration and differentiation were identified through social network analysis; in the second ( Van der Ham *et al.* , 2021 ), rules and coordination mechanisms were identified. In line with ( Van der Ham *et al.* , 2021 ), coordination mechanisms are based on rules, with each type of coordination mechanism requiring different interactions between agents. Taken together, rules and coordination mechanisms can thus account for integration and differentiation.

These case studies showed that tasks are performed mainly across functional silos and that nurses, physicians and coordinators perform integrative tasks. In addition, rules exist predominantly in agents’ minds. Coordination mainly takes place through mutual adjustment and local standardization of work, as defined by Mintzberg (1983) . Long-term schedules for surgeries create open loops ( Munavalli *et al.* , 2017 ), as resources are not scheduled based on actual or future patient demand, potentially destabilizing the system. As the surgery date approaches, several agents in the hospital endeavour to close these loops through mutual adjustment, thereby stabilizing the system.

In our case studies, the observed ways of working seem to have emerged as the result of an individual agent’s action rather than from an explicit organizational design. Central agents coordinate tasks in the absence of authority over others, and largely by means of undocumented working procedures. As Ren *et al.* (2008) point out, such practices may result in vulnerability and instability in the hospital’s performance, leading to critical events. In 2019 the hospital in which our case studies took place – Slingeland Hospital in the Netherlands – decided, independently of the case-study research, to change the way in which surgeries are planned. Since 1 June 2019, a Hospital Planning Centre (HPC) has been responsible for scheduling surgeries and beds for elective and semi-urgent patients. Before that date, surgeries were planned by secretaries in outpatient departments, which are differentiated according to medical discipline.

This change provides us with the opportunity to further explore how the system works; that is, to analyse the extent of the integration and differentiation and how rules and coordination mechanisms shape the hospital’s network structure. Unlike in the two previous case studies ( Van der Ham *et al.* , 2020 , 2021 ), here we also take hospital performance into account. This allows us to shed light on the relationship between integration, differentiation, rules and coordination mechanisms on the one hand and hospital performance on the other.

A key question that arises is whether the introduction of the HPC improved the hospital’s performance. As suggested in Van der Ham *et al.* (2021 ), creating redundancy in the network may increase the robustness of the hospital, altering interaction patterns and the related information processing. In line with Galbraith (1974) , who relates integration to the information-processing capacity of an organization, the introduction of the HPC may have changed the degree of integration, differentiation and ultimately the performance of the hospital. On the other hand, as indicated in Van der Ham *et al.* (2021 ), any intervention may also destabilize the hospital, to the detriment of performance. Instead of evaluating changes following the introduction of the HPC, we focus on exploring changes in terms of integration, differentiation, rules, coordination and performance. By investigating both what has changed and what remains unchanged ( Van Merode, 2021 ; Jullien, 2012 , 2014 ) in the system, we expect to be able to shed light on how a hospital works.

Accordingly, our aim is to explore how the hospital’s social-network structure, coordination mechanisms and performance changed following introduction of the HPC. The research questions are as follows:

1. Did the hospital’s network structure, i.e. integration and differentiation, change following the introduction of the HPC? If so, in what way?
2. Did the rules and coordination mechanisms that account for the network structure change following the introduction of the HPC? If so, in what way?
3. Can changes in the system’s performance be observed following the introduction of the HPC?

## 2. Methods

### 2.1 Setting

This is the third case study in Slingeland Hospital, which is situated in Doetinchem in the Netherlands. Slingeland was selected for its relatively small size, highly rated performance ( Coppa Consultancy, 2012 ) and stable environment. It had 1,843 staff members in 2018 ( Slingeland Hospital, 2019 ) and 1,834 in 2019 ( Slingeland Hospital, 2019 ), including approximately 120 physicians. It serves around 200,000 people in the region and has 350 beds, which is below the average of 450 for Dutch hospitals in general ( Van Hulst and Blank, 2017 ).

In 2018 Slingeland Hospital set up its Integral Capacity Management (ICM) programme. This program focuses on the integrated design, planning, management and safeguarding of all care-related critical capacities, i.e. time, people, space and resources, in order to achieve the desired quality, efficiency and service levels ( Slingeland Hospital, 2020 ). In accordance with this program, the HPC started on 1 June 2019 and is ongoing. The HPC was a new department, with six central planners who are responsible for planning surgeries and beds for surgery patients. Five planners were former outpatient secretaries; the other was a new hire who had not previously worked for Slingeland. In July 2020 the ICM program was converted to a more permanent form, the ICM department, of which the HPC is part. The central planners work in a single office space.

The HPC plans all elective and semi-urgent surgeries that are performed under general anaesthesia. This excludes all eye surgeries and some gynaecology surgeries, which are often performed under local anaesthesia. Urgent surgeries, which take place on the day on which the patient is admitted to the hospital, are planned by the surgeons and secretaries of the outpatient departments, in direct consultation with the Operating Theatre Complex (OTC).

### 2.2 Design

This study was designed to compare the network structure, rules, coordination mechanisms and performance before and after the HPC was introduced. The “before period” was defined as 1 June 2018 to 13 March 2019, the “after period” as 1 June 2019 to 13 March 2020. From the latter date, all but the most urgent surgeries were cancelled due to the Covid-19 pandemic; by choosing this date as a cutoff point, we exclude any potential impact of the pandemic on the study.

**Figure 1:**
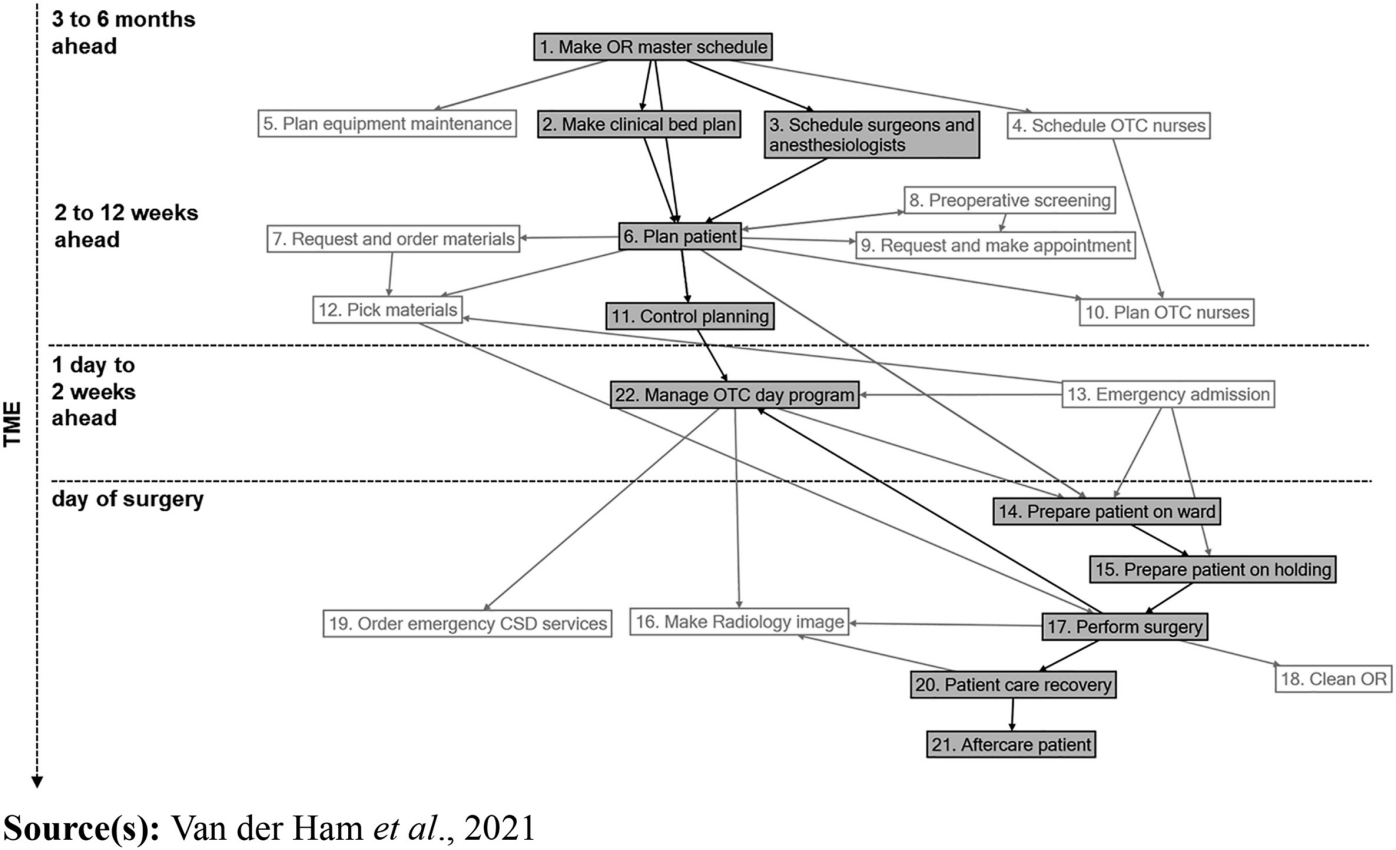
Tasks in scope

To facilitate comparison between the before and after periods, the study design for the third case study is largely the same as for the previous two case studies ( Van der Ham *et al.* , 2020 ) ( Van der Ham *et al.* , 2021 ). Both previous case studies followed the case-study research method ( Yin, 2014 ) and a naturalistic-inquiry approach ( Beuving and De Vries, 2015 ). Data were collected from multiple sources and analysed through data triangulation following a mixed-method approach. The first case study included all departments that contribute to the intake, diagnosis, preparation, surgery or aftercare of surgery patients. The second case study ( Van der Ham *et al.* , 2021 ) focused on planning tasks 1, 2, 3, 6 and 11, and tasks related to coordinating the execution of surgery and the associated preparation and aftercare (tasks 14, 15, 17, 20, 21 and 22) ( Figure 1 ).

In the first case study ( Van der Ham *et al.* , 2020 ), integration and differentiation were described using social network analysis to analyse the network structure. All interactions in which agents engage while performing tasks ( Figure 1 ) were identified, recorded in an Excel database per task and entered into NodeXL ( Smith *et al.* , 2010 ). Specific measures of the social network related to integration and differentiation were analysed, both for the entire network and per task. Density, degree, betweenness centrality and clique overlap were used as indications for integration. A clique exists when all agents in a group are connected. There is clique overlap when agents are part of more than one clique, thereby connecting different cliques. Cliques were explored as potential indicators of differentiation, as groups of highly connected agents may indicate a division of labour. These measures are presented in Table 1 .

The coordination mechanisms and rules studied in the second case study ( Van der Ham *et al.* , 2021 ) refer to the before period. In the present study, the after period was analysed to determine whether a rule still existed or if any new rules had been added. Lapsed rules were recorded, and coordination mechanisms were recorded for each rule (based on Mintzberg, 2012 ) ( Table 1 ). In addition, the performance of the hospital in both the before and the after period was analysed.

In short, in this third case study all concepts as defined in Table 1 were studied for both the before and the after period, with a view to identifying changes following the introduction of the HPC.

### 2.3 Data collection

Data were collected from four different sources: the Hospital Information System (HIS), documentation, observations and interviews. For the before period, data were collected as described in the previous two case studies ( Van der Ham *et al.* , 2020 ) ( Van der Ham *et al.* , 2021 ). For the after period, HIS data and documentation were collected between June and October 2020 specifically for this study. Observations and interviews took place between August and October 2020. As far as possible, staff interviewed in the before period were also interviewed in the after period.

For the total study period between 1 June 2018 and 13 March 2020, HIS data include registrations of surgeries performed in 2018, 2019 and 2020, including the date of surgery, resources involved, timestamps of different stages in the patient’s process, and the nursing wards in which patients stayed before and after surgery. In total, 72 documents were collected between 2018 and 2020, including project plans, management reports, planning schemes, work procedures, emails and internal presentations. Of these, 64 date from the before period and 8 from the after period.

Planning and controlling activities were observed during 19 observation days between 2018 and 2020 (18 in the before period, 1 in the after period). Four observations, between March and June 2019, focused on observing preparations for the HPC. The 19 observations took place, variously, in three outpatient departments, three nursing departments, the holding area, during three surgeries in the operating room (OR), in the recovery area, with the OTC day coordinator, in the preoperative screening department, during two planning meetings and twice at the workplace of two (future) central planners and at the HPC. During each observation, the activities of the hospital staff were observed and unplanned informal conversations took place, as staff explained the tasks in which they were engaged. The sequence of events for each observation, together with relevant parts of the conversations, was reported in an observation report.

There were more observations in the before period (18) than in the after period (1). This is because planning activities were observed in the centrally located HPC in the after period, whereas multiple outpatient departments were observed in the before period to gain the required understanding of the planning activities. Additionally, two people who were observed in the before period were interviewed in the after period.

In the before period, between March 2018 and June 2019, 25 interviews were held with 23 different people. In the after period, between August 2020 and January 2021, 19 interviews were held with 19 people. A total of 14 people were interviewed in both the before and after periods. Two people were observed in the before period and interviewed in the after period. People who were interviewed only in the before period were involved in tasks that fall outside the scope of this study, but who were relevant to our earlier case study ( Figure 1 ). People who were interviewed only in the after period were new to a role that had been performed by another person in the before period. For each interview, a topic list was prepared, including questions on agents, interactions, rules, coordination and performance. All interviews were recorded and transcribed ad verbatim with the consent of the respondents.

### 2.4 Data analysis

As in the first case study ( Van der Ham *et al.* , 2020 ), the network structure, i.e. the agents and their mutual interactions, was constructed per task ( Figure 1 ). The social-network analysis based on data from 2017 to 2018 ( Van der Ham *et al.* , 2020 ) was updated to reflect the entire before period; for example, the number of agents involved in the tasks was updated. Subsequently, the social network was constructed for the after period. Measures for integration and differentiation were analysed for both the before and after periods.

All 44 data sources for rules and coordination mechanisms for the after period (i.e. all documents, observations and interviews) were structured into five data matrices. One data matrix was constructed for each of the tasks 1, 2 and 3; one for tasks 6 and 11 together; and one for tasks 14, 15, 17, 20, 21 and 22 together. These five matrices were constructed for the before period in the second case study, drawing on 94 qualitative sources.

We then analysed whether a rule still existed in the after period and whether it was applied hospital wide or locally, i.e. within one particular department/group of people or by one person. Any new rules were added and rules that had been abolished were recorded as such. In addition, we analysed whether rules ( *R* ) or the output of applying the rule (O) were recorded in a document or in the HIS, or whether they existed only in the minds of hospital staff. Coordination mechanisms ( Mintzberg, 2012 ) were recorded for all rules in the after period, based on the definitions of the coordination mechanisms as presented in Table 1 .

Next, we listed all performance indicators mentioned in the 138 data sources (documents and interviews) from the before and after periods. Performance indicators that could be calculated on the basis of available HIS data were included in this study ( Table 1 ). We recorded which and how many sources mentioned each performance indicator before the introduction of the HPC. In addition, we recorded whether the indicator or desired changes were described in qualitative or quantitative terms. The values for these performance indicators were calculated for both the before and after periods.

A focus group session involving the manager of the ICM Department (who served as the OTC capacity planner in the before and the after period) and a central planner was held to validate the recorded performance indicators. This entailed discussing whether the performance indicators reflected the performance in reality. The manager and central planner considered all performance indicators to be valid.

## 3. Results

### 3.1 Network structure

Following the introduction of the HPC, the number of agents involved in the network decreased from 526 to 514 ( Table 2 ). Table 3 shows that in the after period, fewer OR nurses, secretaries and ward nurses participated in the network, and one less manager was involved. On the other hand, the number of surgeons and/or surgeon assistants increased and six central planners were added.

The number of cliques decreased by 10%, from 7,692 to 6,909. This change is mainly attributable to the fact that in the after period, surgeries were performed more frequently by the same OR team. While there were 6,660 unique OR teams, i.e. cliques, in the before period ( Table 4 ), this figure dropped to 5,881 in the after period. Network parameters associated with integration, i.e. density, degree and betweenness centrality barely changed ( Table 2 ).

Table 3 reveals a change in the network position of managers. In the before period, one cluster manager was also manager of the OTC, a position taken over in the after period by an OTC team leader. The new OTC manager had a higher degree (25) and betweenness centrality (311) in the after period than the average degree (11) and centrality (4) of the cluster managers in the before period. Table A1 in Appendix 1 shows that the OTC manager became involved with planning surgeries ( task 6 ), whereas in the before period the cluster manager was not involved.

The position of the OTC capacity planner changed as well. In the after period, her degree and centrality decreased by 78% and 88%, respectively. Although she was formally employed as OTC capacity planner until July 2020, her network position and tasks changed earlier, i.e. during the after period. After the HPC was introduced, the OTC capacity planners’ tasks shifted to the OTC manager and central planners. In addition, the centrality of the outpatient secretaries, surgeons, OTC team leaders and OTC day coordinator decreased substantially.

With the fourth highest betweenness centrality, the central planners hold a central position in the network. Compared to their former position as secretaries of the outpatient department, their higher degree and betweenness centrality mean they are now better connected to many agents. The OTC day coordinator is most central, followed by nurse anaesthetists and recovery nurses. This central position is mainly explained by the fact that, on the day of a surgery, they interact with all agents involved in performing surgeries, i.e. OR nurses, nurse anaesthetists, surgeons, anaesthesiologists and ward nurses, who transfer patients to and from the OTC. In addition, on the day before a surgery the OTC day coordinator interacts with the central planners and outpatient secretaries on planning tasks.

The neurosurgeon had the highest betweenness centrality in both the before and the after period. This is because the neurosurgeon is the only surgeon who visits patients in nursing ward N1; all other surgery patients stay in other nursing wards. This makes him the only agent in the entire network who interacts with the nurses of nursing ward N1. As shown by the social networks in Appendix 3 , his network position is therefore relatively central.

### 3.2 Network structure per task

The social networks per task ( Appendix 2 ) and Table 4 reveal that the network structure for tasks 1, 2, 6 and 11 changed in the after period. The OR master schedule (task 1) came to be created by a denser network, illustrated by an increased density from 0.3 to 0.9, and 50% fewer agents ( Figure B1 ), with outpatient secretaries and two cluster managers no longer involved in this task. Clique overlap decreased by 67%, which can be explained by the fact that the number of agents participating in multiple cliques decreased from six to two.

The clinical bed plan (task 2) was taken over by the central planners, interacting with nursing-ward team leaders ( Figure B2 ). With the number of cliques rising from one to three in the after period, density decreased by 8%.

For patient planning (task 6), density increased by 83% and clique overlap increased by 500%. Figure B4 in Appendix 2 shows that in the after period, six planners had connections with all surgeons and outpatient secretaries, instead of with one OTC capacity planner who coordinated planned surgeries.

Planning control (task 11) involved 72% fewer agents in the after period, i.e. 15 instead of 54, increasing the density of the network from 0.2 to 0.7. Clique overlap rose from 6% to 40%.

With regard to scheduling surgeons and anaesthesiologists (task 3), ten networks were not connected in either the before or the after period ( Figure B3 , Appendix 2 ). With more surgeons in the after period, the density for task 3 increased by 31%. Tasks performed on the day of surgery barely changed in terms of density and the number of cliques; any changes were in the number of agents involved. The number of cliques for performing surgeries (task 17) decreased by 12%, as there were fewer unique OR teams in the after period.

### 3.3 Rules and coordination

Table 5 shows that after the introduction of the HPC, the total number of rules decreased by 7%. Although there were 8% more hospital-wide rules, rules applied locally decreased by 38%. This is largely attributable to tasks 6 and 11, for which local rules were cut by 83%. Of 314 rules from the before period, 63 rules (20%) changed in the after period.

For task 1, the number of rules decreased by 6% and 23 of 53 rules were changed. During the after period, as of 1 January 2020, the OR master schedule was restructured, taking bed availability in the nursing wards into account. In addition, the HPC took more initiative and control over the process of returning or granting extra sessions, and rules related to this were changed accordingly.

A similar change can be observed for task 2, for which the number of rules decreased by 27% and 19 of 51 rules were changed. In the before period, the clinical bed plan was informally controlled by a nursing ward secretary. In the after period, the HPC was given planning and control over the beds for nursing wards A2, N2 and the Short Stay Department.

Nine new rules were introduced and 20 rules changed for patient planning (task 6) and planning control (task 11). A number of local rules from the before period were standardized into one hospital-wide rule to be implemented by the HPC. For example, in the after period the HPC set surgery dates approximately two weeks ahead of the surgery, whereas in the before period some outpatient departments set these dates in an earlier stage (e.g. at the time of the patient’s visit to the outpatient department) while others did not. Semi-urgent surgeries came to be treated by the HPC as “regular” surgeries, i.e. not scheduled between planned surgeries, but planned as though they were elective surgeries, even though they have to take place on shorter notice. The maximum planned utilization of an OR session decreased from 105% to 100%, a rule strictly applied by the HPC. Additionally, in the after period the HPC had to plan all patients in the HIS instead of in paper planning documents.

Rules did not change for scheduling surgeons and anaesthesiologists (task 3); i.e. the majority of rules remained local. For tasks 14 to 22 the rules did not change either (with the exception of one), and these remained mostly hospital-wide rules. In both the before and after periods, rules primarily existed in people’s minds (85%) and to a lesser extent in documents (31% before and 33% after) or in the HIS.

Turning to coordination mechanisms, standardization of work and mutual adjustment remained the most common mechanisms. There was no direct supervision, as the central planners hold no formal authority over other agents. Standardization of output and of norms was low. For task 1, standardization of output decreased by 33%, mutual adjustment decreased by 8% and standardization of work increased by 12%. This can be explained by the restructuring of the OR master schedule by the OTC manager and the OTC capacity planner during the after period. The clinical bed plan was restructured in line with the OR master schedule, and central control of the HPC led to 34% less mutual adjustment and 17% less standardization of work. For planning and control tasks 6 and 11, no large changes were observed with regard to coordination mechanisms. Likewise, for tasks 3 and 14 to 22, coordination mechanisms and all tasks on the day of surgery remained largely unchanged.

### 3.4 Performance indicators

Before introducing the HPC, Slingeland Hospital envisaged changes in 17 performance indicators ( Table 1 ). Table 6 shows that the expected changes for each performance area were mostly specified in qualitative terms. Only waiting list length and resource utilization were reported in quantitative terms in both the before and the after period, although the desired or expected change was not specified in advance. Several improvements were envisaged for performance indicators related to how well processes were planned. Most frequently identified in the before period were improved planning of surgery time and less overutilization of ORs, lateness and cancellations (mentioned in six documents and four interviews), as well as less variability (mentioned in six documents and three interviews).

While the number of surgeries performed stayed roughly the same, the average waiting list length increased by 21%, from 5.8 to 7 weeks on average. Figure D1 in Appendix 4 shows that this increase started in week 37 (9 September) of 2019, three months after the HPC was introduced. The waiting list length stabilized at the start of 2020. In addition, the ORs and OR sessions were utilized slightly less (3%) and beds were utilized 11% more. The latter can be attributed to the fact that the number of beds was reduced by 14% in the after period.

The main change with regard to process quality and control was an 88% increase in cancellations. Figure D2 in Appendix 4 shows the number of cancellations classified according to the reason for cancellation. The increase can largely be explained by administrative factors. In the after period, surgeries were entered “incorrectly” in the system 172% more often than in the before period, and surgeries for which the surgery date was unknown increased by 5250%. This was attributed to the fact that rule 167 required planners to plan all surgeries in the HIS, including those for which the surgery date was not final and the patient had not yet been informed of the date. Leaving out these two administrative factors, the number of cancellations increased by 15%.

Lateness in the OR, i.e. the average number of late ORs and the percentage of days on which surgeries ran late, decreased by 2% and 4% respectively. The difference between the planned and actual surgery time increased by 7%.

Variability changed little on average, with the exception of waiting list variability, which increased by 19% ( Table 6 ). Table 6 also shows that the maximum values for OR utilization, OR session utilization and bed utilization all decreased by 9%–20%.

## 4. Discussion

This study identified changes that followed the introduction of an HPC in terms of network structure (i.e. integration and differentiation), rules, coordination and hospital performance. Based on density, integration increased mainly on the task level for planning tasks in which the OR master schedule is set (task 1), surgeries and beds are planned (task 6) and planning is controlled (task 11). Central planners took on a central position for the main planning tasks 6 and 11, and the OTC manager took on a more central position, thus contributing to integration. Differentiation based on medical discipline remained after the introduction of the HPC and was observed for tasks 3 and 6, which involve the planning of surgeons and surgeries. Network-wide integration did not change substantially.

Rules came to be applied less locally, i.e. rules from the before period became the hospital-wide standard or new hospital-wide rules were set. However, rules remained mostly in people’s minds; there was little increase in documented rules.

Coordination mechanisms remained largely the same, primarily involving standardization of work and mutual adjustment in both the before and after periods.

With regard to performance, the number of beds decreased and bed utilization increased. Peak values in resource utilization also decreased. However, not all performance indicators that had been identified as desirable prior to the introduction of the HPC improved. A notable increase in waiting list length and cancellations, as well as increased variability of OR utilization and waiting list length, was observed following the introduction of the HPC.

In sum, the overall network structure and coordination mechanisms remained largely the same, but integration and certain rules changed for specific planning tasks. It is striking that the hospital’s performance did not seem to improve substantially and even decreased in some respects. Although the relationship between the observed changes cannot be established by this study alone – and nine months may be too short a period to assess the hospital-wide impact of the HPC – a number of possible explanations related to integration, differentiation and coordination can be identified.

First, the fact that the hospital-wide network structure barely changed, rules remained largely undocumented and coordination mechanisms remained the same suggests that integration “emerges” rather than being consciously designed or developed. This is also illustrated by the fact that the 17 performance indicators used in this study were not documented and quantified prior to the introduction of the HPC but rather manually extracted from 138 different sources for the purposes of this study. This echoes Lawrence and Lorsch’s (1967 , p. 12) assertion that “when the environment requires both a high degree of subsystem differentiation and a high degree of integration, integrative devices will tend to emerge”. Differentiation leads to the development of interdependent subsystems, giving rise in turn to a “felt need for joint decision making” ( Lawrence and Lorsch, 1967a , b ). Following this line of reasoning, the HPC may have emerged from local agents making use of their autonomy; as Tummers *et al.* (2006) point out, agents with high autonomy tend to adapt organizational structures to fit their own work needs.

Second, those elements that did not change after the introduction of the HPC are clearly persistent, if not immutable. The HPC continued to plan surgeries for specific groups of patients, excluding non-surgical patients and certain surgery patients, and did not control the surgeons’ schedules. According to Ludwig *et al.* (2010) , a hospital’s efficiency is most likely to improve when *all* departments cooperate rather than some, as at Slingeland. Further, the HPC did not appear to take patient demand into account more than the outpatient departments had done in the before period. Long-term schedules, of surgeons in particular, remained largely unchanged, with waiting list length determined mainly by the available capacity. While patient inflow remained approximately the same in the before and after periods, the maximum utilization of OR sessions was reduced, leading to an increase in waiting list length. In addition, surgeons and outpatient secretaries, who have direct and first contact with patients, came to hold a less central position in the after period. This appears to be in conflict with Galbraith (1974) and Lawrence and Lorsch (1967a , b) , who indicate that the organizational structure (i.e. the degree of integration and differentiation) should, for optimal performance, be tuned to the environment. Moreover, the unchanged long-term schedules still created open loops ( Munavalli *et al.* , 2017 ) which were closed by mutual adjustment, as in the before period ( Van der Ham *et al.* , 2021 ). Although six planners (instead of one OTC capacity planner) were available to close the loops, the causes of instability, e.g. the multitude of rules and the open loops, did not seem to have been reduced but instead had merely shifted from the outpatient departments and the OTC capacity planner to the HPC. The increased length of the waiting list and number of cancellations suggest that the network remained unstable or indeed become even more unstable.

Given that hospital performance did not increase after the introduction of the HPC, a more conscious approach to achieving integration and differentiation appears to be needed. Such an approach should take into account the fact that differentiation in hospitals is inextricably linked to medical and nursing tasks requiring specific knowledge and experience. This goes hand in hand with a certain degree of professional autonomy. More specifically, the HPC should form part of a redesign of the entire system, including network structure, rules and coordination mechanisms. The ways of working observed in Slingeland Hospital show similarities with the “horizontal coordination” in certain Japanese companies which are reputed for the quality of their products and efficient production systems. According to Aoki (1990) , horizontal coordination involves collective learning and knowledge sharing based on informal and mostly verbal communication. This is most effective in dynamic environments in which all agents have a good understanding of the whole work process and have internalized the organizational goals. In the case of the HPC, stronger lateral relations appear to have been created ( Aoki, 1986 ; Galbraith, 1974 ), but whether there is collective learning, knowledge sharing and full understanding of organization-wide goals remains unclear.

Starting with organization-wide goals, we would propose that the management of Slingeland Hospital first explicitly specifies the desired performance indicators. Literature pertaining to performance indicators ( Van der Ham *et al.* , 2018 ) and hospital planning ( Demeulemeester *et al.* , 2013 ; Hulshof *et al.* , 2012 ; Zhu *et al.* , 2019 ; Munavalli *et al.* , 2020 ) could be used to formulate the expected changes in performance. These goals should then be discussed and disseminated hospital wide. Regularly monitoring, evaluating and communicating changes in the network structure, rules, coordination mechanisms and performance would facilitate expansive learning, a process in which agents produce new forms of work activity ( Engeström, 2001 ) based on feedback. Job rotation ( Aoki, 1990 ) could enhance agents’ understanding of hospital-wide processes and prevent them from identifying too narrowly with the specific subsystems in which they work. This is in line with Mintzberg (2012) , who called for more mutual adjustment and standardization of norms in hospitals. The use of incentives could also motivate agents to act in the interests of the hospital-wide goals ( Aoki, 1990 ).

Next, the organizational-design concepts of integration, differentiation and centralization require further elaboration, particularly in practice. The word “centre” in HPC implies that centralization of planning was intended. In Mintzberg’s (1983) view, however, centralization is related to decision power. The lack of direct supervision at Slingeland means that planning tasks seem instead to be concentrated *without* power: planners have been grouped together and given the competency to perform planning tasks without wielding actual decision power. From the perspective of horizontal coordination ( Aoki, 1990 ), agents involved in controlling operations may not require formal decision power; after all, agents can influence decision-making processes irrespective of their formal positions ( Van Merode *et al.* , 2004a , b ). Concentrating the planners – i.e. further centralizing them in the network structure by involving them in planning processes from start to end – may effectively increase their influence.

For effective horizontal coordination, Slingeland Hospital would also do well to pay more attention to differentiation. This means explicitly specifying differentiated tasks, encouraging central planners, outpatient secretaries, surgeons and anaesthesiologists to share goals and information, and allowing them to “stop the line” whenever they see issues that might lead to problems. In the event of any further organizational changes, the hospital management could enhance the chances of success by explicitly indicating what organizational model underpins them.

Similar to the previous two case studies ( Van der Ham *et al.* , 2020 , 2021 ), this was an exploratory study. As with any case study, the findings, if not broadly generalizable, can be used for “analytic generalization” ( Yin, 2014 , p. 14); in this case, to help clarify the connections between performance, network structure, integration, differentiation, rules and coordination mechanisms in general. First, our findings suggest that the concept of integration as defined in social network and organizational theory needs to be developed further. Integration in Slingeland Hospital appeared to increase for some tasks when analysed through the lens of social network metrics, but arguably did not change when taking decision power into account ( Lawrence and Lorsch, 1967a , b ; Mintzberg, 1983 ).

Second, some network metric changes we identified seem to be attributable to factors other than the introduction of the HPC, such as the decreased number of unique OR teams and the position of the neurosurgeon. As Beuving and De Vries (2015) point out, network performance can only be understood when accompanied by an understanding of what happens between the agents in the network. Based on these findings we argue that social network analysis alone is not sufficient to explain changes in integration and differentiation. The analysis should be supplemented by the studying the rules and coordination mechanisms, in order to understand how a network works.

Third, the concept of horizontal coordination is worth exploring further, but the cost of intensive interaction between multiple agents and time-consuming learning processes will pay off only in volatile and dynamic environments ( Aoki, 1990 ), for which a more hierarchical model is ineffective. More research is thus required on the dynamics of the hospital’s environment. In addition, Takt Time Management should be considered in relation to horizontal-coordination models. This synchronizes the desired time between units of output with the customer’s demand, with a view to stabilizing the system ( Munavalli *et al.* , 2017 ). A multi-agent system including horizontal coordination, such as that designed by Munavalli *et al.* (2020) , could be used to further develop hospital-wide coordination, integration and differentiation.

This study has identified a number of changeable and persistent connections in a hospital network’s structure, rules, coordination mechanisms and performance, providing important input for future studies, not only in hospital settings but also in other organizations that provide healthcare on a regional or national level (e.g. supply chain partners of hospitals). With the accessibility and affordability of healthcare systems under ever more pressure, particularly since the Covid-19 pandemic, the relationships between integration, differentiation, coordination and the stability of healthcare networks and organizations will only become more important in the future.

## Figures and Tables

**Table 1:** Definition of concepts

Concept	Definition
Integration	The coordination and alignment of tasks, thus achieving “unity of effort among the various subsystems in the accomplishment of the organization's task” ( Lawrence and Lorsch, 1967a , b , p. 4)
Differentiation	The “state of segmentation of the organizational system into subsystems” ( Lawrence and Lorsch, 1967 , pp. 3–4)
*Social network analysis*	
Node	An agent
Tie	A communication link between two agents via email, text message, telephone or face-to-face
Clique	A set of agents who are all connected to one another
Density	The number of ties a set of agents has in relation to the number of possible ties they can have
Clique overlap	The percentage of agents who are members of more than one clique for a specific task
Degree	The number of ties of one agent
Betweenness centrality	The number of times a node (agent) lies on the shortest path between other nodes (agents)
*Rules and coordination mechanisms*	
Rule	A defined, accepted or agreed way of performing tasks, which includes what is done, how it is done and what is allowed and what is not allowed
Mutual adjustment	An agent interacts with other agents about a rule, i.e. what it entails or how to apply it in a specific situation or the application of the rule requires interaction
Direct supervision	A rule is set and monitored by people with formal authority
Standardization of work processes	Rules result from specified or programmed working processes
Standardization of output	Rules include specified output in terms of predetermined standards for services or performance
Standardization of skills	Rules include specified skills and knowledge
Standardization of norms	Rules result from a common culture or ideology and specify norms for behaviour
*Performance indicators*	
Patient inflow	The number of surgery orders within a specified period
Number of surgeries	The number of surgical cases performed
Number of elective/non-elective surgeries	The number of planned/(semi-)urgent surgeries
Surgery time	The time between starting a surgery, i.e. when patient positioning and/or skin preparation starts, to when the surgery is completed
Planned versus realized surgery time	The difference between realized and planned surgery time
Overutilization operating room (OR)	The total number of operating room days that finished after 4 pm divided by the total number of regular operating room days on which elective surgeries took place
Number of late operating rooms	The number of operating rooms that finish after 4 pm.
Cancellations	The number of cancelled surgeries
Waiting list length	Average number of weeks necessary for operating on all patients on the waiting list, given the number of OR sessions allocated to a medical discipline
OR session utilization	The total amount of time surgical patients are present in the OR, divided by the amount of allocated OR session time. An OR session is a time slot that is allocated to a specific medical discipline on a specific weekday and OR.
OR utilization	The total amount of time surgical patients are present in the OR during regular working hours between 8 am and 4 pm, divided by the total amount of OR time during these working hours (8 h)
Bed utilization	The average number of beds used for a patient in a nursing department divided by the number of available beds
Number of beds	The number of beds in the nursing departments
Variability OR utilization	The degree to which OR utilization deviates from the average OR utilization
Variability OR session utilization	The degree to which OR session utilization deviates from the average OR session utilization
Variability bed utilization	The degree to which bed utilization deviates from the average bed utilization
Variability waiting list length	The degree to which waiting list length deviates from the average waiting list length

**Table 2:** Network parameters of the overall network in the before and after periods

Network parameter	Before 1 June 2019	After 1 June 2019	Change
Number of agents	526	514	−2%
Number of ties	32.002	31.703	−1%
Density	0.23	0.24	4%
Number of cliques	7,692	6,909	−10%

Agent parameter	Lowest	Average	Highest	Lowest	Average	Highest	Lowest	Average	Highest
Degree	1	122	411	2	124	402	100%	2%	−2%
Betweenness centrality	0	240	5,611	0	233	4,728	N/A	−3%	−16%

**Table 3:** Network metrics per role in the before and after periods

Role	Number of agents in network			Average degree			Average betweenness centrality		
	Before	After	Change	Before	After	Change	Before	After	Change
Anaesthesiologist	12	12	0%	159	152	−4%	147	133	−10%
Central planner	N/A	6	N/A	N/A	90	N/A	N/A	686	N/A
Cluster manager	3	1	−67%	11	2	−82%	4	0	−100%
Holding nurse	4	4	0%	272	268	−2%	601	549	−9%
Nurse anaesthetist	31	31	0%	372	372	0%	1481	1452	−2%
OR nurse	61	51	−16%	129	135	5%	41	44	7%
OTC capacity planner	1	1	0%	99	22	−78%	2,155	267	−88%
OTC day coordinator	3	3	0%	198	189	−5%	3,507	2,736	−22%
OTC manager	0	1	N/A	N/A	25	N/A	N/A	311	N/A
OTC team leader	11	11	0%	32	33	2%	111	13	−88%
Recovery nurse	10	10	0%	246	245	0%	1949	1937	−1%
Secretary	41	34	−17%	17	17	−3%	60	1	−99%
Surgeon or assistant surgeon	58	63	9%	148	132	−11%	432	360	−17%
Ward nurse	291	286	−2%	99	103	4%	44	46	5%

**Table 4:** Network metrics for each task

	Task	Number of agents			Number of ties			Density			Number of cliques			Clique overlap				
		Before	After	Change	Before	After	Change	Before	After	Change	Before	After	Change	Before		After		Change
1	Make OR master schedule	28	14	−50%	110	82	−26%	0.3	0.9	209%	2	2	0%	6	21%	2	14%	−67%
2	Make clinical bed plan	4	9	125%	6	33	450%	1	0.9	−8%	1	3	200%	N/A	N/A	6	67%	N/A
3	Schedule surgeons and anaesthesiologists	65	60	−8%	195	218	12%	0.09	0.12	31%	9	9	0%	0	0%	0	0%	N/A
6	Plan patient	79	88	11%	315	715	127%	0.1	0.2	83%	42	50	19%	1	1%	6	7%	500%
11	Control planning	54	15	−72%	224	75	−67%	0.2	0.7	356%	2	5	150%	3	6%	6	40%	100%
14	Prepare patient on ward	280	278	−1%	12.109	11914	−2%	0.3	0.3	0%	248	248	0%	31	11%	31	11%	0%
15	Prepare patient in holding	282	277	−2%	1071	1054	−2%	0.03	0.03	2%	278	273	−2%	4	1%	4	1%	0%
17	Perform surgery	162	157	−3%	8,653	8,183	−5%	0.7	0.7	1%	6,660	5,881	−12%	146	90%	147	94%	1%
20	Patient care recovery	290	284	−2%	2,383	2,374	0%	0.06	0.06	4%	278	273	−2%	10	3%	10	4%	0%
21	Aftercare of patient	383	386	1%	12,630	12,736	1%	0.2	0.2	−1%	171	176	3%	221	58%	222	58%	0%
22	Manage OTC day program	201	187	−7%	600	555	−8%	0.03	0.03	7%	1	1	0%	N/A	N/A	N/A	N/A	N/A

**Table 5:** Rules and coordination mechanisms per task in the before and after periods

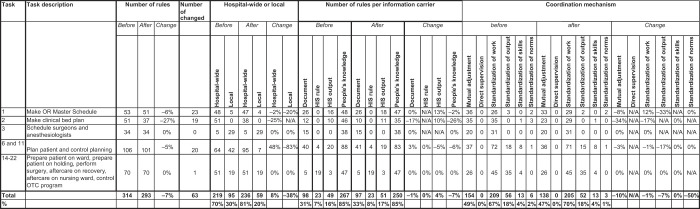

**Table 6:** Performance indicators in the before and after periods

Performance indicator	Description	Indicator specification		Number of sources			Value			Unit of measure
		Quantitative	Qualitative	Total	Document	Interview	Before	After	Change	
*1. Surgeries performed*										
Patient inflow	The average number of surgery orders made per week		X	3	1	2	192	183	−5%	Number of surgery cases
Number of surgeries	Total number of surgical cases performed						7,888	7,470	−5%	Number of surgeries
Number of elective and non-elective surgeries	Elective or non-elective/emergency cases performed (%) included are all inpatient surgical cases excluded are cases performed at separate OR locations						83%	83%	0%	%
Surgery time	The sum of surgery times of all surgeries, with surgery time being the time starting when patient positioning and/or skin preparation can begin to when the surgery is completed						8,845	8,604	−3%	Hours
*2. Process quality and control*										
Planned vs realised surgery time	Average % difference between realised and planned surgery time		X	10	6	4	29%	31%	7%	%
Overutilization OR	The total number of operating room days that finished after 4 pm divided by the total number of regular operating room days on which elective surgeries took place						27%	26%	−4%	%
Number of late ORs	The average number of operating rooms that finish after 4 pm.						2.01	1.96	−2%	Number of ORs
Cancellations	Number of surgeries that were cancelled						992	1863	88%	Number of surgeries
*3. Variability*										
Variability OR utilization	Standard deviation of OR utilization		X	9	6	3	19%	20%	5%	%
	Minimum OR utilization: The lowest OR utilization for an OR on a weekday						5%	7%	40%	%
	Maximum OR utilization: The highest OR utilization for an OR on a weekday						134%	107%	−20%	%
Variability OR session utilization	Standard deviation of OR session utilization						7%	8%	14%	%
	Minimum OR utilization: The lowest OR session utilization for an OR on a weekday						66%	57%	−14%	%
	Maximum OR utilization: The highest OR session utilization for an OR on a weekday						111%	100%	−10%	%
Variability bed utilization	Average standard deviation of bed utilization divided by the average bed utilization and minimum and maximum OR utilization						29	29	0%	Number of beds
	Minimum bed utilization						1	1	0%	Number of beds
	Maximum bed utilization						172	157	−9%	Number of beds
Variability waiting list	Average standard deviation of waiting list length divided by the average waiting list length						3.1	3.7	19%	Weeks
	Minimum waiting list length						1	1.3	30%	Weeks
	Maximum waiting list length						15.9	19.3	21%	Weeks
*4. Waiting list*										
Waiting list length	Average number of weeks necessary for operating on all patients on the waiting list, given the number of OR sessions allocated to a medical discipline	X		7	5	2	5.8	7	21%	Weeks
*5. Resource utilization*										
OR session utilization	The total amount of time surgical patients are present in the OR, divided by the amount of allocated OR session time *x* 100%. An OR session is a time slots that is allocated to a specific medical discipline on a specific weekday and OR.	X		5	4	1	87%	84%	−3%	%
OR utilization	The total amount of time surgical patients are present in the OR during regular working hours between 8 am and 4 pm, divided by the total amount of OR time during these working hours (8 h)						77%	75%	−3%	%
Bed utilization	The average number of beds used for a patient in a nursing department divided by the number of available beds, for nursing departments A2, N2 and the daycare/Short stay department						76%	84%	11%	%
*6. Resource availability*										
Number of beds	The number of beds in nursing departments A2, N2 and the daycare/Short stay department		X	6	4	2	96	83	−14%	Number of beds

## Appendix

The appendices are available online for this article.
