# Supplementary material for: Exploring changes in integration, differentiation, rules, coordination and performance following the introduction of a hospital planning centre: a case study
Source: J Health Organ Manag. 2022 Apr 8;36(9):158–78. doi: 10.1108/JHOM-10-2021-0375 (PMC10424640; doi:10.1108/JHOM-10-2021-0375)
Supplement: Supplementary file 1 [file jhealthorganmanag-36-0158-g009.docx]

**Appendix A: Involved agents per task**

**Table A1:** Tasks and agent types involved in these tasks

**Appendix B: Social networks**

***B1. The entire social network***

**
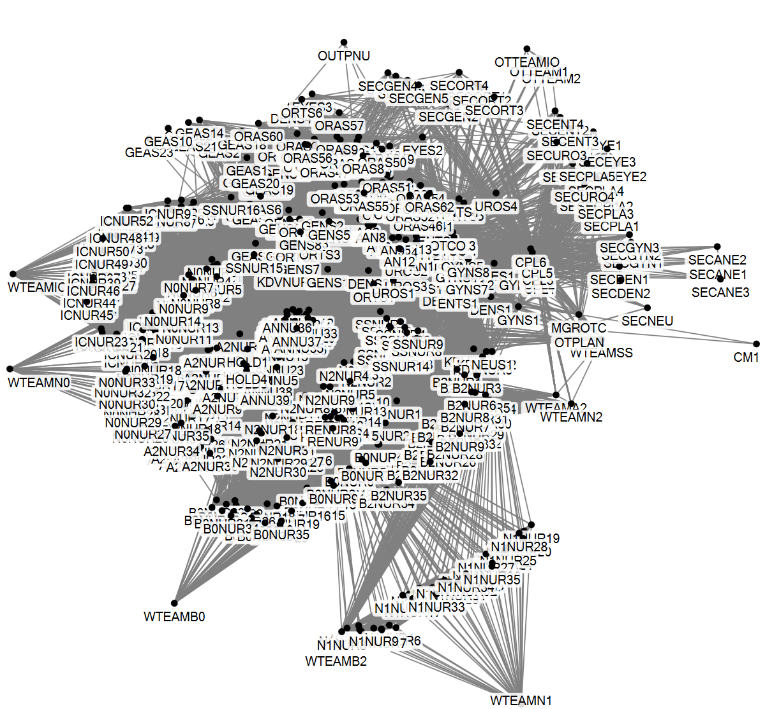
**
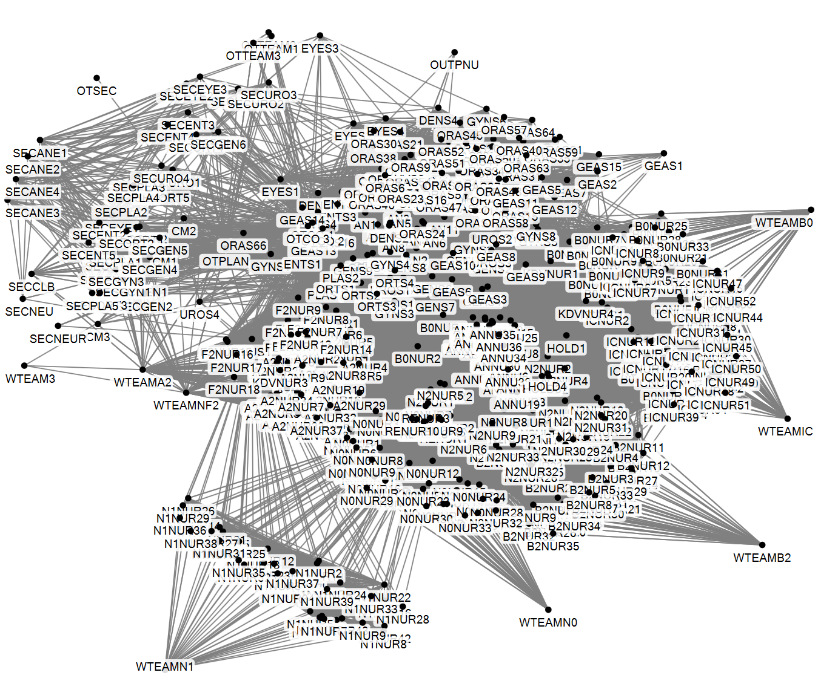
**Before period** **After period**

**Figure B1:** Before and after social networks and metrics for the entire network

***B2. Task 1: Make OR master schedule***


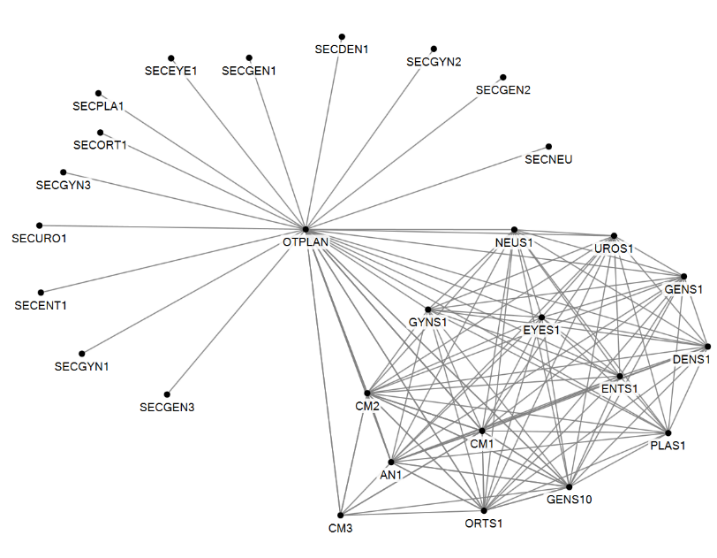
**Before** **period** **After period**


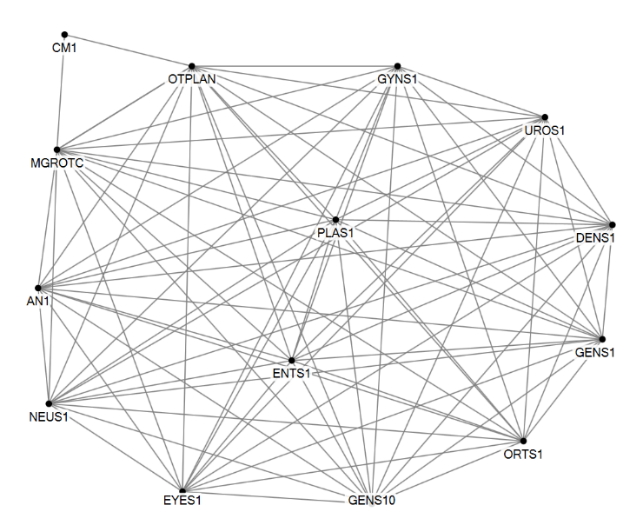

**Figure B2:** Before and after social networks and metrics for task 1

***B3. Task 2: Make clinical bed plan***

**Before** **period** **After period**


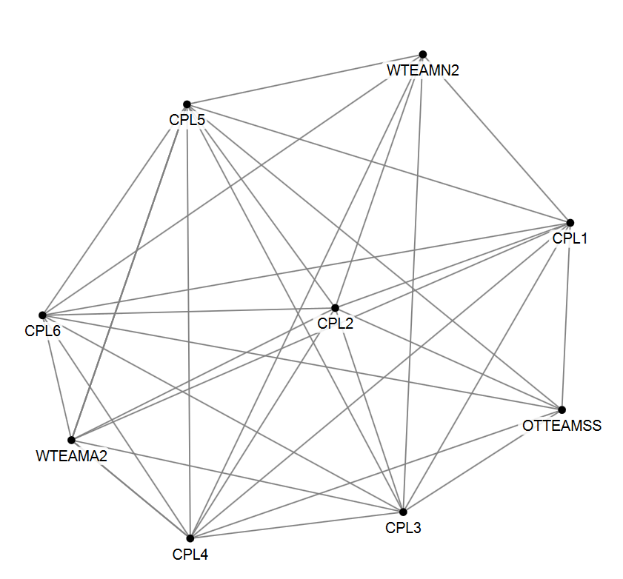


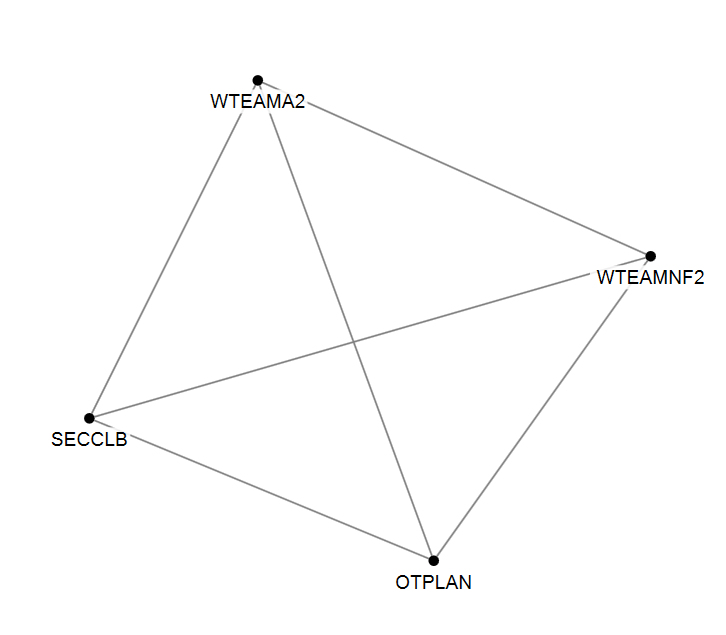

**Figure B3:** Before and after social networks and metrics for task 2

***B4. Task 3: Schedule surgeons and anesthesiologists***

**Before** **period** **After period**


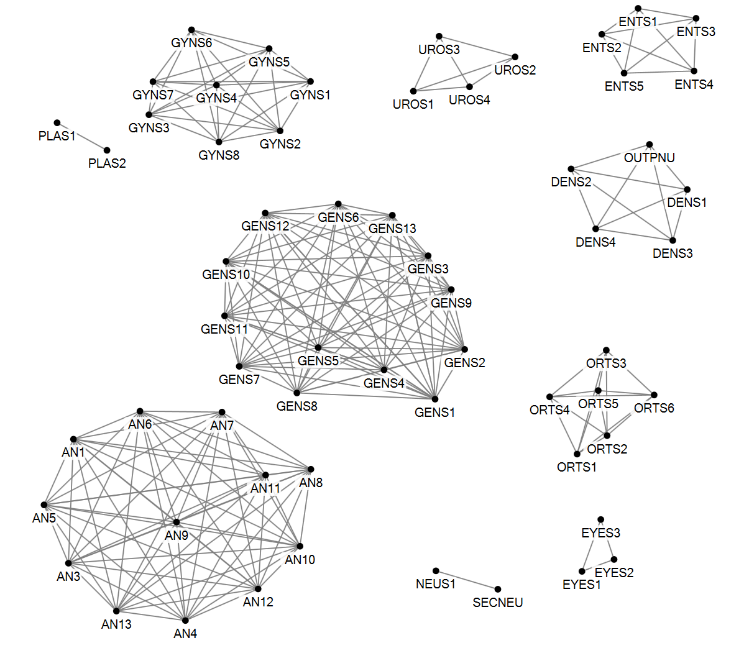

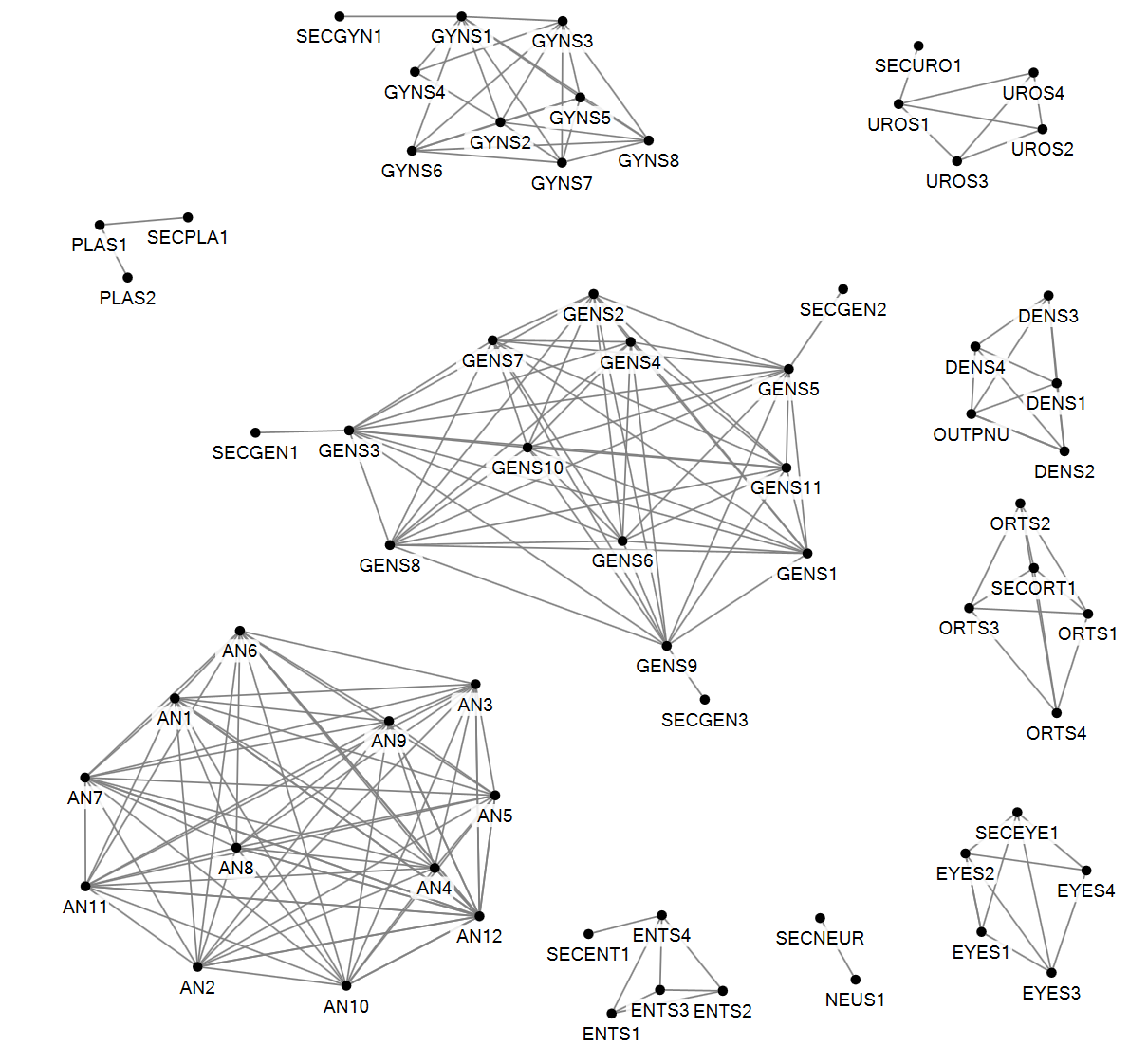

**Figure B4:** Before and after social networks and metrics for task 3

***B5. Task 6: Plan patient***

**Before** **period** **After period**


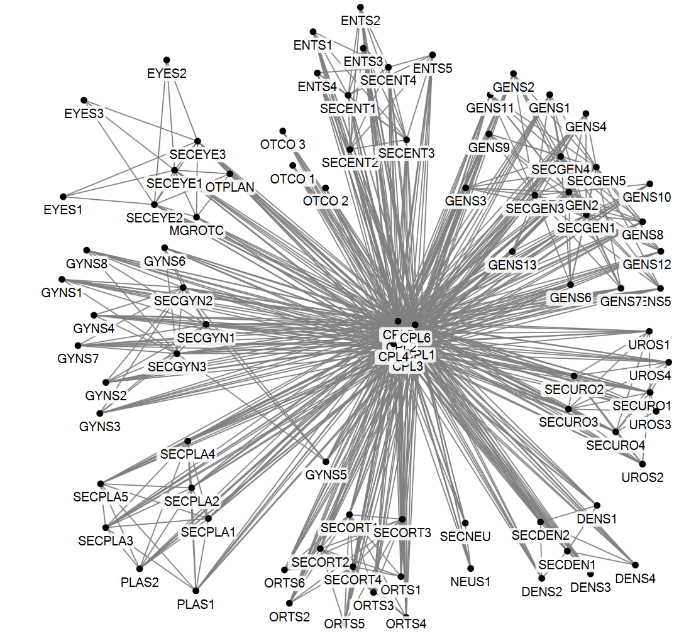

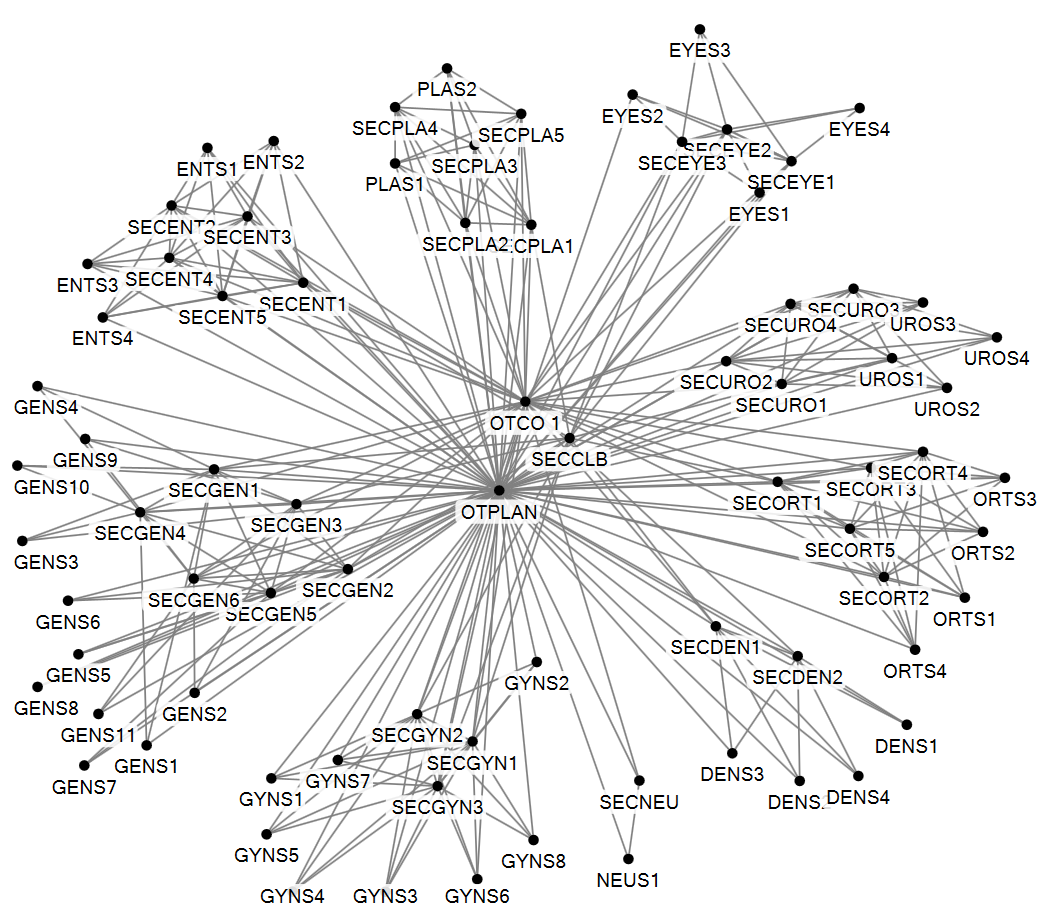

**Figure B5:** Before and after social networks and metrics for task 6

***B6. Task 11: Control planning***

**Before** **period** **After period**


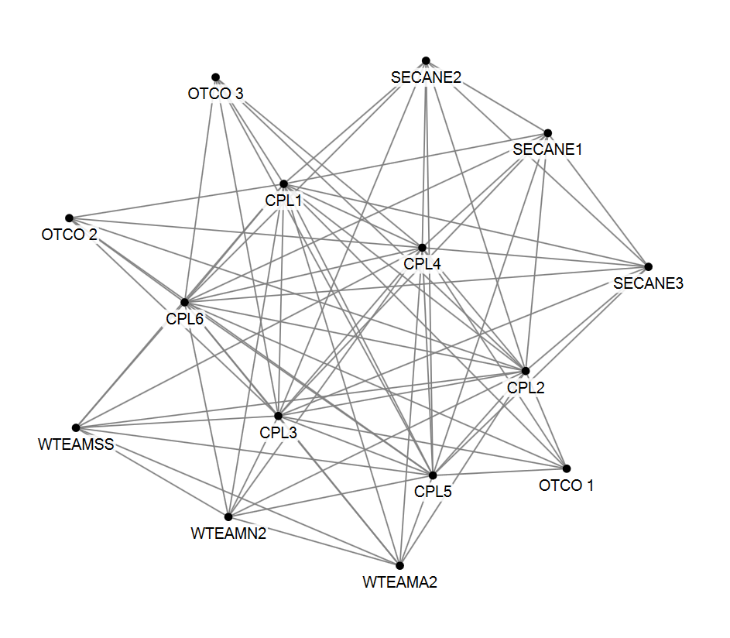


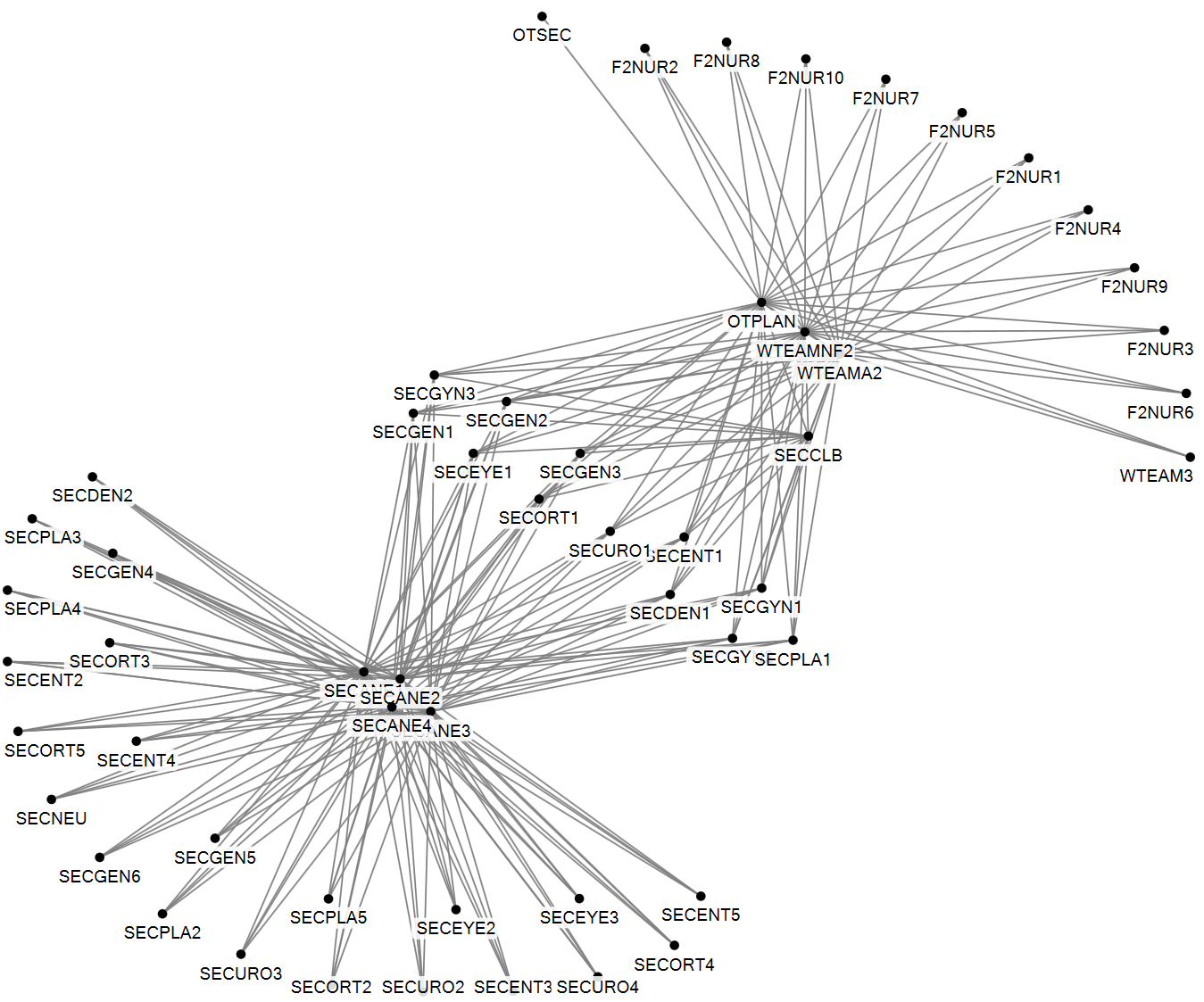

**Figure B6:** Before and after social networks and metrics for task 11

***B7. Task 14: Prepare patient on ward***


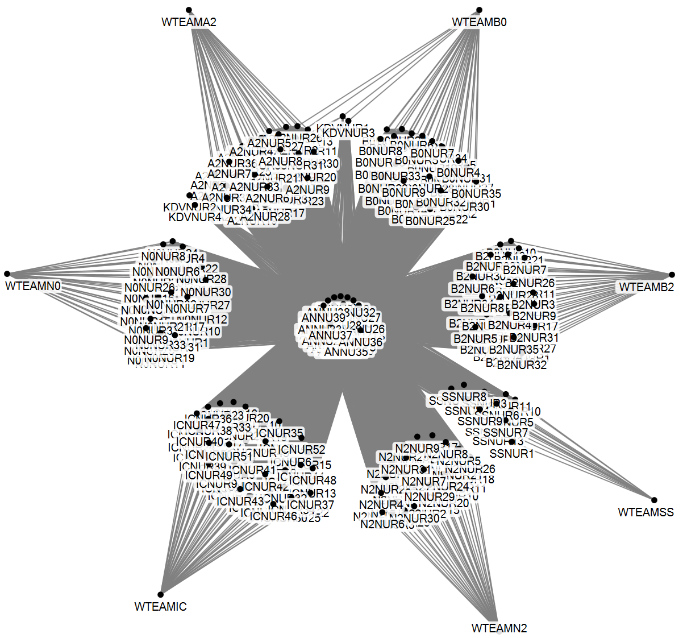
**Before period** **After period**


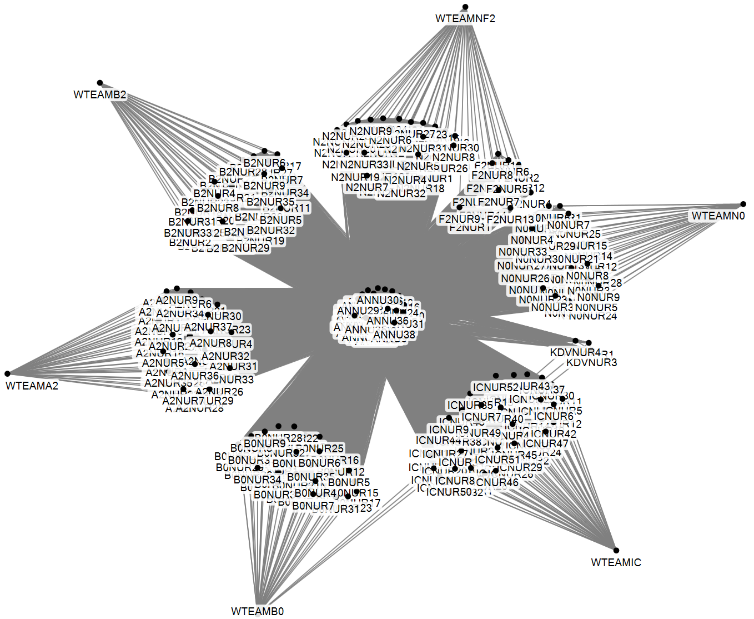

**Figure B7:** Before and after social networks and metrics for task 14

***B8. Task 15: Prepare patient on holding***

**Before period** **After period**


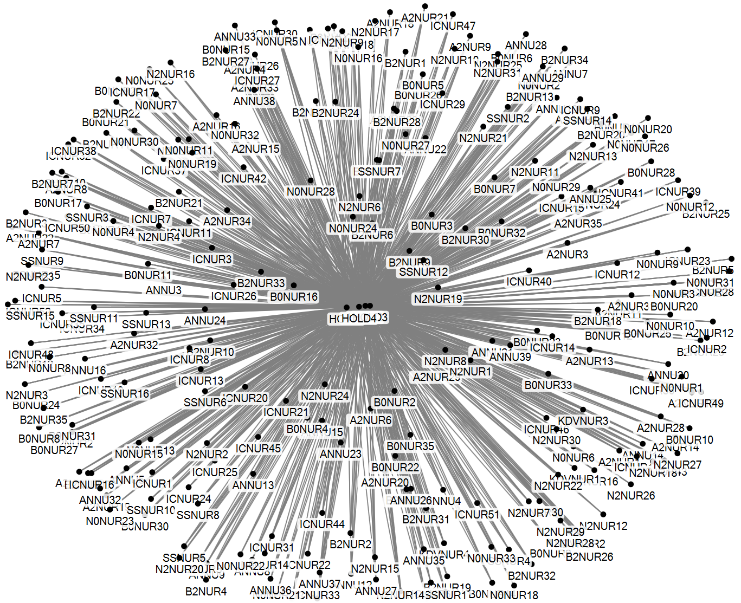

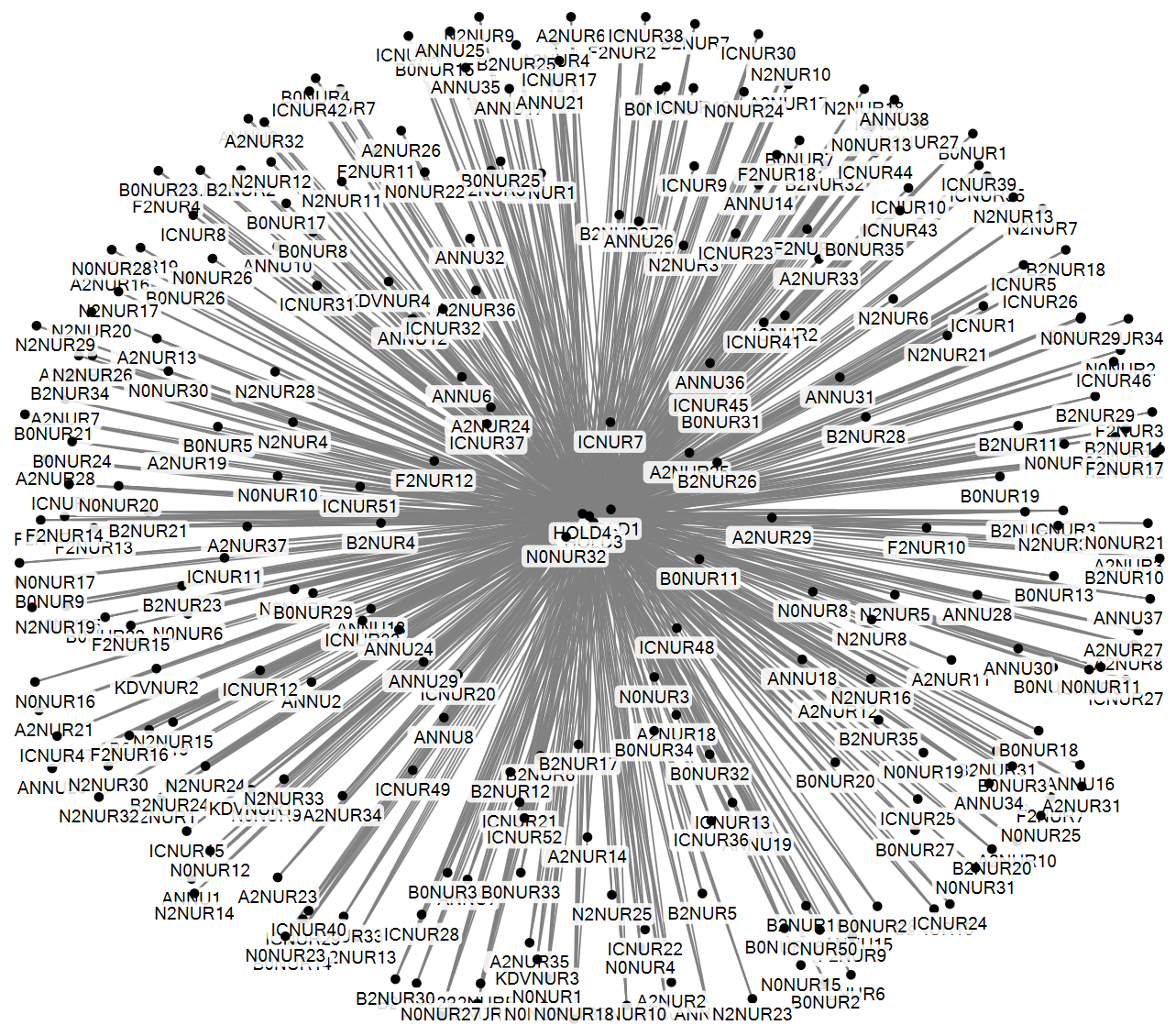

**Figure B8:** Before and after social networks and metrics for task 15

***B9. Task 17: Perform surgery***

**Before** **period** **After period**


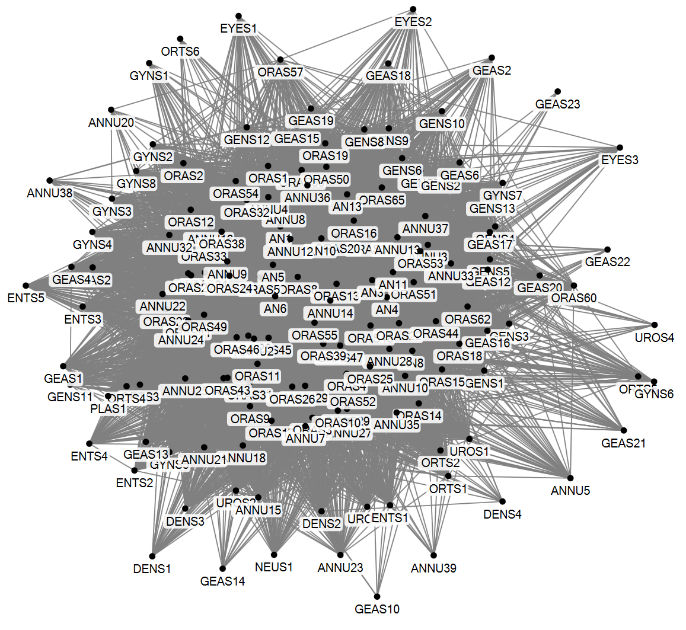

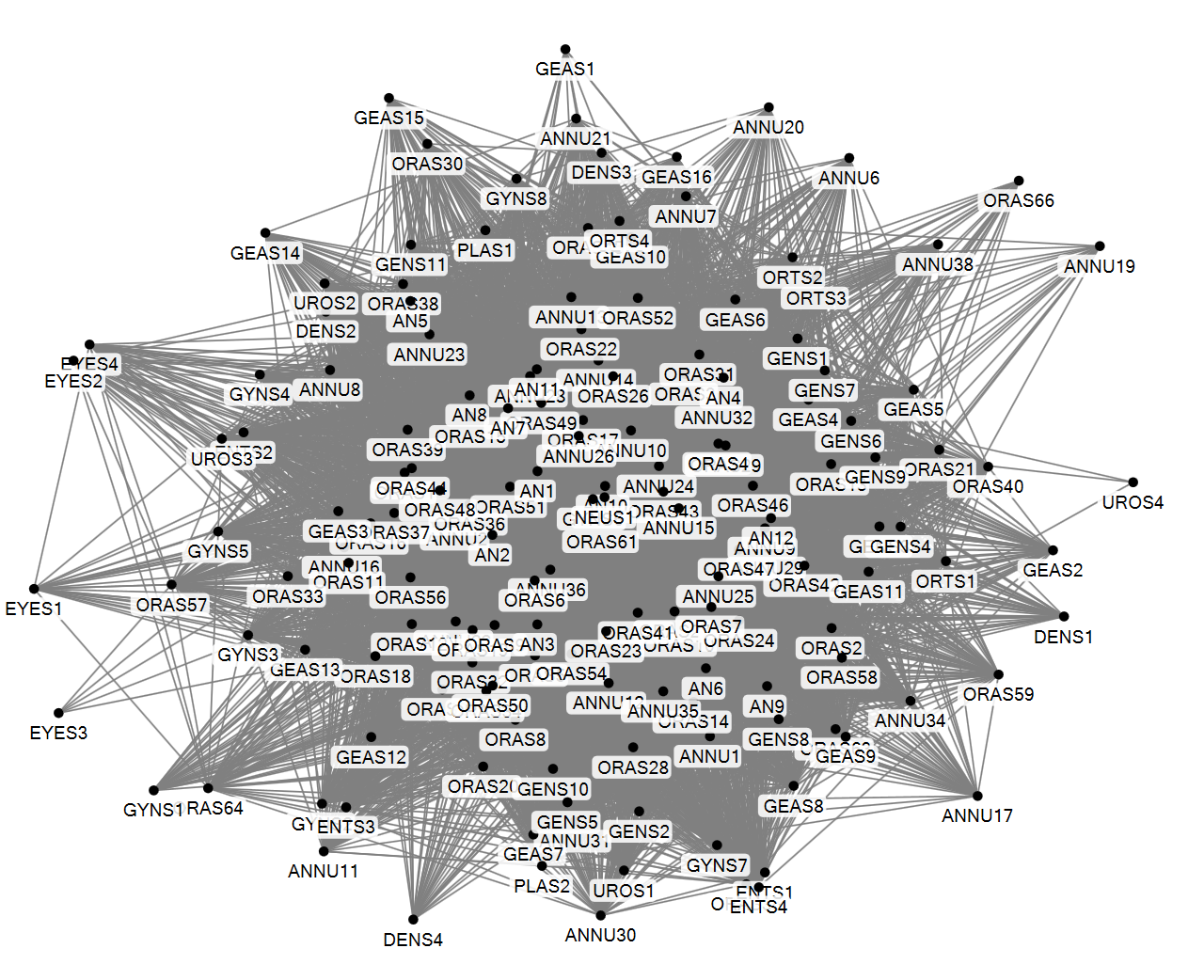

**Figure B9:** Before and after social networks and metrics for task 17

***B10. Task 20: Patient care Recovery***

**Before** **period** **After period**


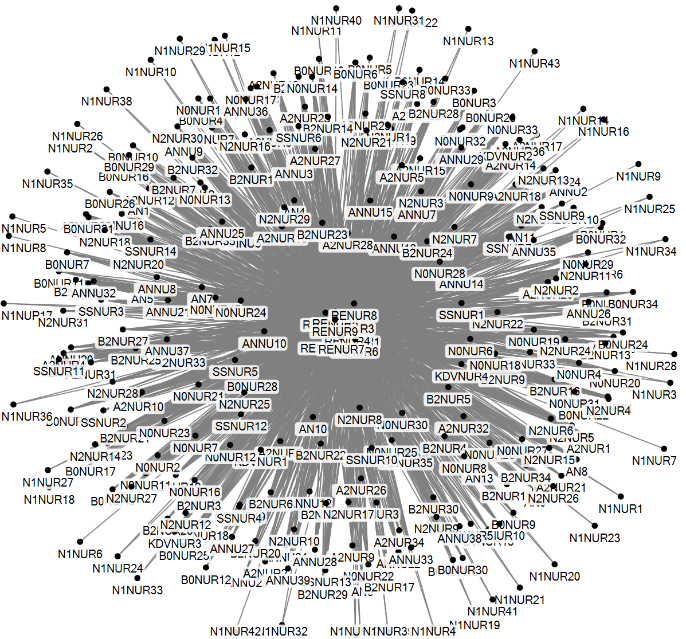

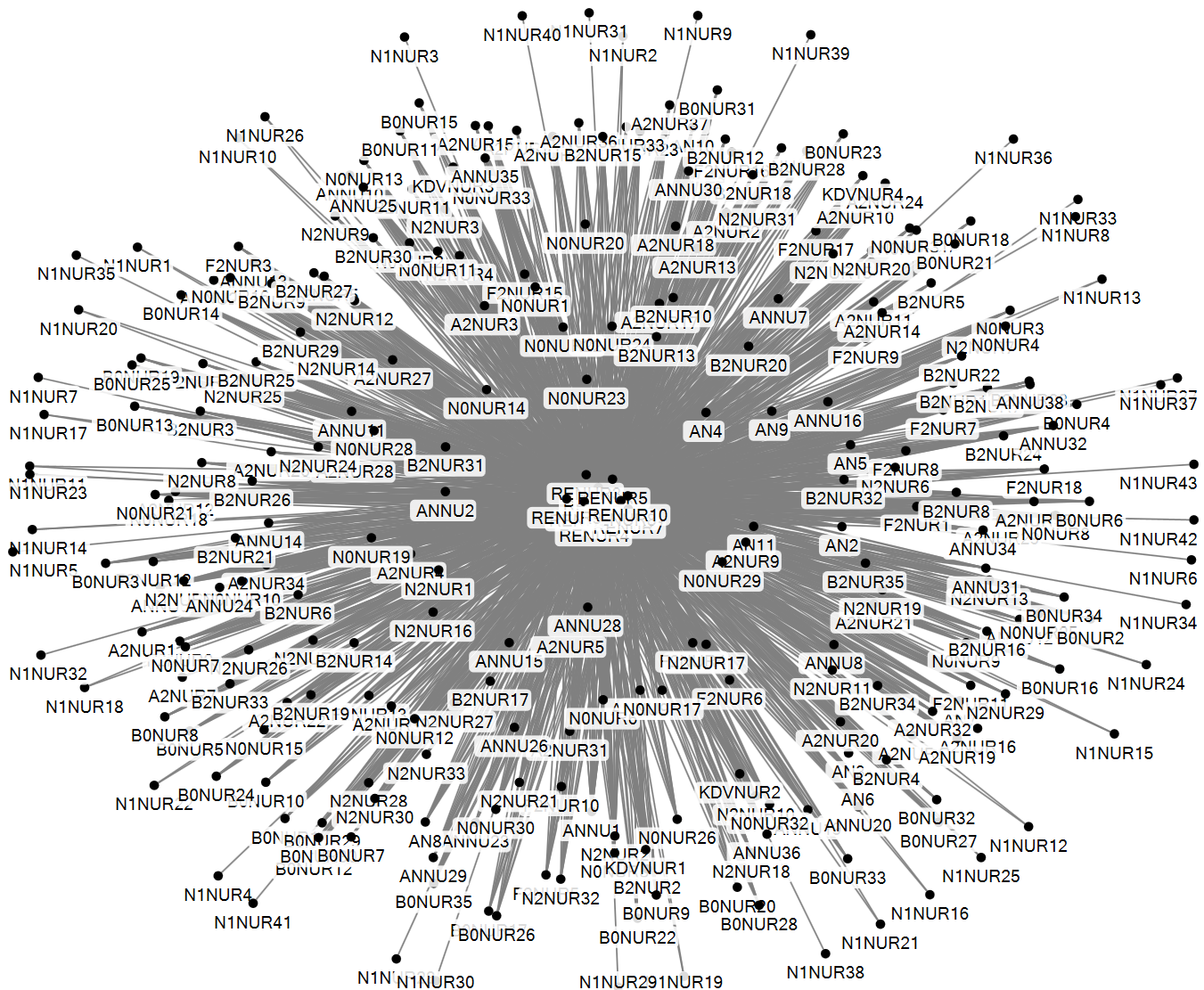

**Figure B10:** Before and after social networks and metrics for task 20

***B11. Task 21: Aftercare patient***

**Before** **period** **After period**


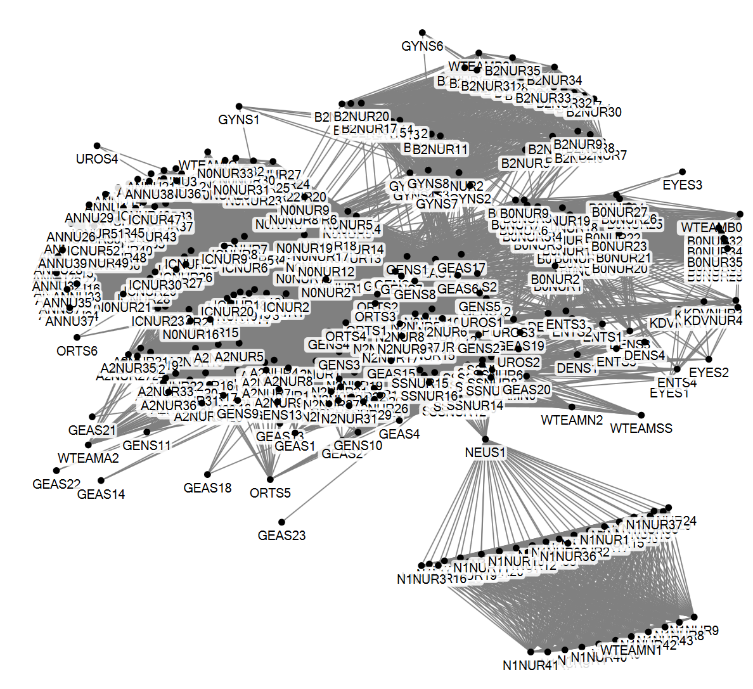


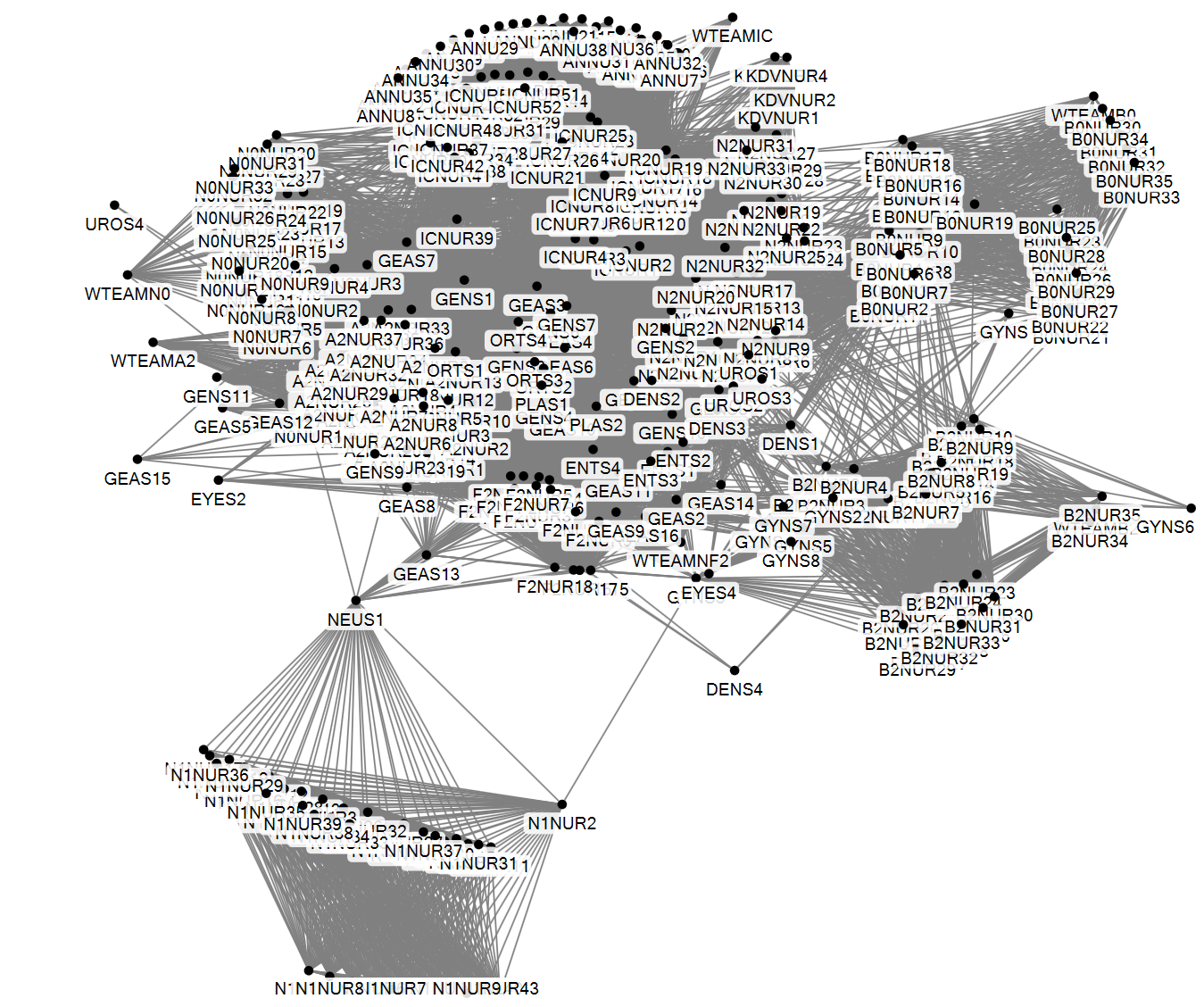

**Figure B11:** Before and after social networks and metrics for task 21

***B12. Task 22: Manage OTC day program***

**Before** **period** **After period**


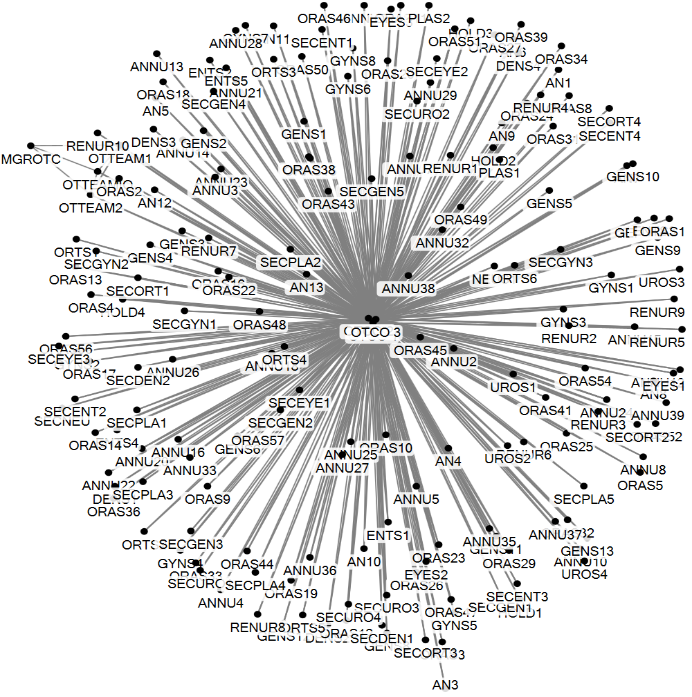


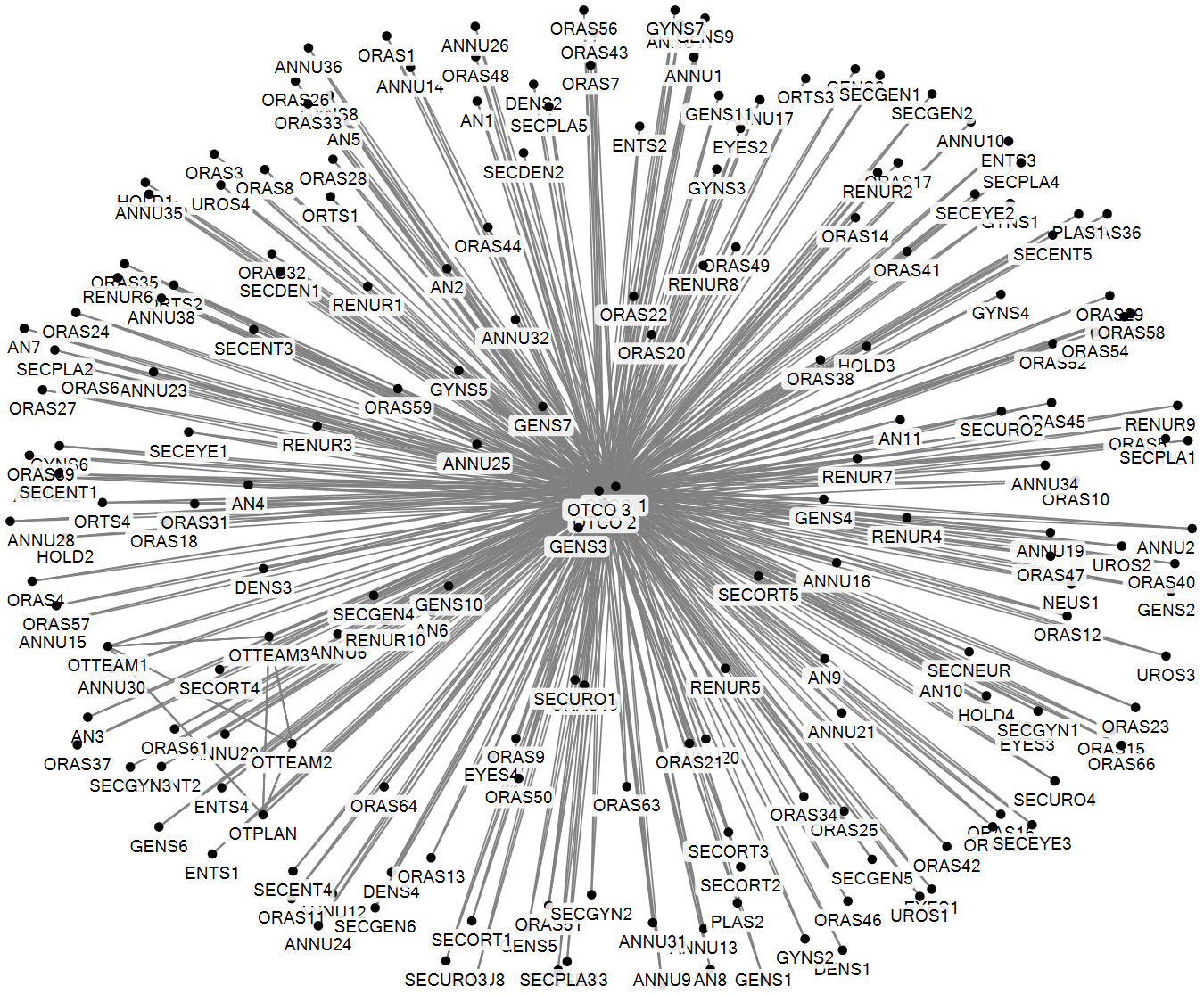

**Figure B12:** Before and after social networks and metrics for task 22

***B13.*** ***Description of agent codes in social networks***

**Appendix C: Rules and coordination mechanisms**

All rules in the before and after period are presented. A rule applied in the before period either remains to exist in the after period, does not exist in the after period, is new or is adapted, in which case the adaptations to the rule are described.

***Task 1: Make OR Master Schedule***

***Task 2: Make Clinical Bed Plan***

***Task 3: Schedule surgeons and anesthesiologists***

***Tasks 6 and 11: Plan patient and control planning***

**Tasks 14,15,17,20,21 and 22: Prepare patient on ward, holding, perform surgery and aftercare on recovery and nursing ward, control OTC program**

**Appendix D: Performance indicators**


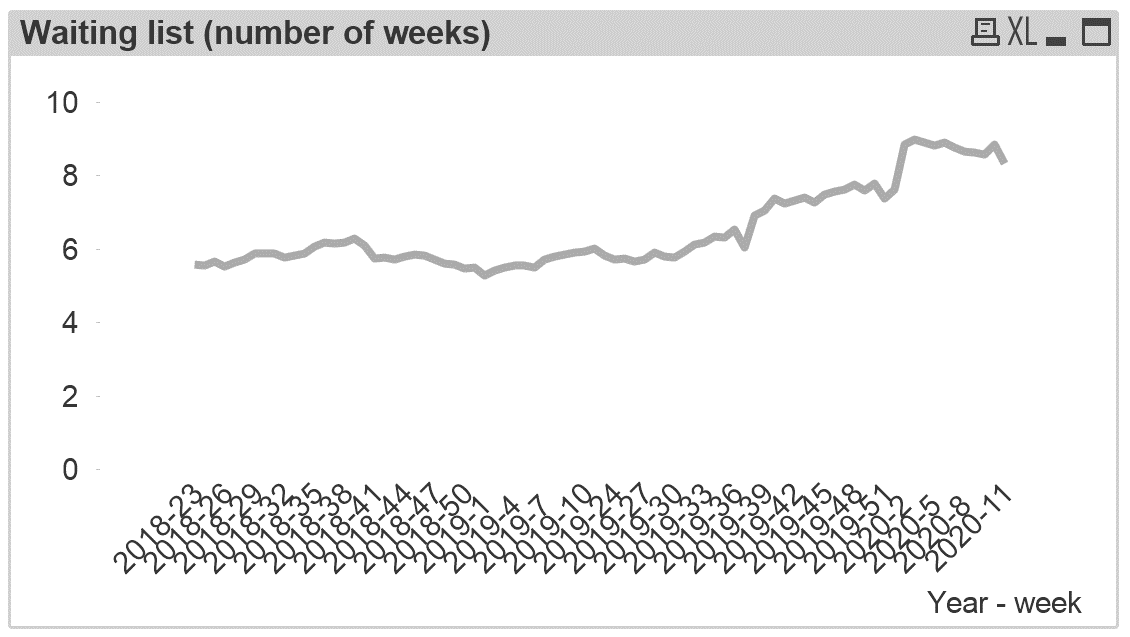


start HPC

**Figure D1:** Waiting list development


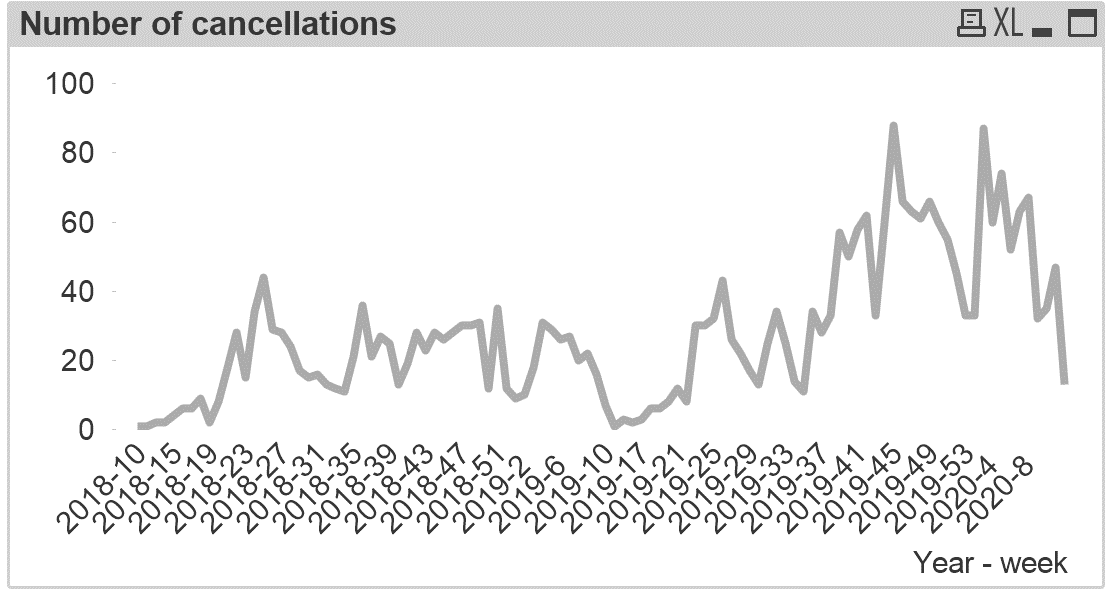


start HPC

**Figure D2:** Number of cancellations

**Table D1:** Registered reasons for cancellation in the before and after period
